# Darolutamide in Japanese patients with metastatic hormone‐sensitive prostate cancer: Phase 3 ARASENS subgroup analysis

**DOI:** 10.1002/cam4.70029

**Published:** 2024-11-11

**Authors:** Motohide Uemura, Hiroaki Kikukawa, Yasuhiro Hashimoto, Hiroji Uemura, Atsushi Mizokami, Masashi Kato, Hisashi Matsushima, Takeo Kosaka, Motonobu Nakamura, Satoshi Fukasawa, Matthew R. Smith, Bertrand Tombal, Maha Hussain, Fred Saad, Karim Fizazi, Cora N. Sternberg, E. David Crawford, Haruka Kakiuchi, Masanao Akiyama, Rui Li, Iris Kuss, Heikki Joensuu, Hiroyoshi Suzuki

**Affiliations:** ^1^ Department of Urology Osaka University Hospital Osaka Japan; ^2^ Department of Urology National Hospital Organization Kumamoto Medical Center Kumamoto Japan; ^3^ Department of Urology Hirosaki University School of Medicine and Hospital Hirosaki Japan; ^4^ Department of Urology Yokohama City University Medical Center Yokohama Japan; ^5^ Department of Urology Kanazawa University Hospital Kanazawa Japan; ^6^ Department of Urology Nagoya University Hospital Nagoya Japan; ^7^ Department of Urology Tokyo Metropolitan Police Hospital Tokyo Japan; ^8^ Department of Urology Keio University Hospital Tokyo Japan; ^9^ Department of Urology National Hospital Organization Kyushu Cancer Center Fukuoka Japan; ^10^ Prostate Center and Division of Urology Chiba Cancer Center Chiba Japan; ^11^ Genitourinary Oncology Program Massachusetts General Hospital Cancer Center and Harvard Medical School Boston Massachusetts USA; ^12^ Division of Urology, IREC Cliniques Universitaires Saint‐Luc, Université Catholique de Louvain Brussels Belgium; ^13^ Division of Hematology‐Oncology, Department of Medicine, Robert H. Lurie Comprehensive Cancer Center Northwestern University Feinberg School of Medicine Chicago Illinois USA; ^14^ Department of Urology University of Montreal Hospital Center Montréal Québec Canada; ^15^ Department of Cancer Medicine Institut Gustave Roussy, University of Paris–Saclay Villejuif France; ^16^ Department of Medicine Englander Institute for Precision Medicine, Meyer Cancer Center, Weill Cornell Medicine, New York‐Presbyterian Hospital New York New York USA; ^17^ Department of Urology University of California San Diego School of Medicine La Jolla California USA; ^18^ Bayer Yakuhin, Ltd Osaka Japan; ^19^ Bayer Yakuhin, Ltd Tokyo Japan; ^20^ Bayer HealthCare Pharmaceuticals Inc. Whippany New Jersey USA; ^21^ Bayer AG Berlin Germany; ^22^ Orion Corporation Espoo Finland; ^23^ Department of Urology Toho University Sakura Medical Center Sakura Japan; ^24^ Present address: Department of Urology Iwase General Hospital Fukushima Japan; ^25^ Present address: Funabashi Municipai Medical Center Funabashi Japan

**Keywords:** darolutamide, efficacy, Japanese, metastatic hormone‐sensitive prostate cancer, safety

## Abstract

**Background:**

In the global ARASENS study (NCT02799602), darolutamide plus androgen‐deprivation therapy (ADT) and docetaxel significantly reduced risk of death by 32.5% versus placebo plus ADT and docetaxel (hazard ratio [HR] 0.68; 95% confidence interval [CI] 0.57–0.80; *p* < 0.0001), with a favorable safety profile in patients with metastatic hormone‐sensitive prostate cancer (mHSPC). We investigated outcomes in Japanese participants.

**Methods:**

Patients were randomized 1:1 to oral darolutamide 600 mg twice daily or placebo, plus ADT and docetaxel. The primary endpoint was overall survival.

**Results:**

The Japanese subgroup comprised 148 patients (darolutamide 63, placebo 85). In the Japanese versus overall population, more patients were aged ≥75 years (darolutamide/placebo 35%/22% vs. 16%/17%) and had body mass index <25 kg/m^2^ (78%/79% vs. 46%/43%), The ECOG performance status 0 (92%/88% vs. 72%/71%), de novo mHSPC (95%/97% vs. 86%/87%), and Gleason score ≥8 (94%/92% vs. 78%/79%). Median treatment duration was 43.3/15.4 months for darolutamide/placebo. The overall survival HR for darolutamide versus placebo was 0.91 (95% CI 0.50–1.64), despite 85% of patients in the placebo group receiving subsequent life‐prolonging therapy. Darolutamide prolonged time to castration‐resistant prostate cancer (HR 0.31; 95% CI 0.17–0.55). Treatment‐emergent adverse event incidences were generally similar between groups. Adverse events known to be associated with docetaxel (e.g., neutropenia) were more frequent in the Japanese versus overall population.

**Conclusion:**

In conclusion, efficacy outcomes showed positive trends for darolutamide plus ADT and docetaxel in Japanese patients with mHSPC, consistent with the overall population, despite higher risk factors. The combination was well tolerated, with no new safety signals in Japanese patients.

## Abstract 2

In Japanese patients with metastatic hormone‐sensitive prostate cancer (mHSPC) who participated in the global phase 3 ARASENS study, darolutamide in combination with androgen‐deprivation therapy (ADT) and docetaxel showed favorable trends in efficacy and tolerability over placebo plus ADT and docetaxel, with no new safety concerns. The outcomes were consistent with the findings in the overall study population and support the use of darolutamide in combination with ADT and docetaxel in Japanese patients with mHSPC as a new standard of care.

## 1 INTRODUCTION

Patients with metastatic hormone‐sensitive prostate cancer (mHSPC) may be initially asymptomatic, but most progress to metastatic castration‐resistant prostate cancer (CRPC), typically within 1–3 years on androgen‐deprivation therapy (ADT) alone.^1^, ^2^, ^3^, ^4^ Development of castration resistance is associated with reduced survival, increased burden of disease, and deteriorating quality of life.^5^, ^6^, ^7^ Therefore, the goals of early treatment for mHSPC are to prolong survival and delay disease progression with minimal impact on patients' quality of life.

Over the past few years, the international standard of care for mHSPC has evolved to combine ADT with either docetaxel or an androgen receptor pathway inhibitor (ARPI; abiraterone acetate, enzalutamide, or apalutamide),^8^, ^9^, ^10^ based on significantly increased survival and delayed progression versus ADT alone.^2^, ^3^, ^4^, ^11^, ^12^, ^13^, ^14^, ^15^, ^16^, ^17^, ^18^, ^19^, ^20^, ^21^, ^22^ In 2022, the phase 3 ARASENS and PEACE‐1 studies demonstrated significant survival benefits from ADT in combination with an ARPI (darolutamide or abiraterone acetate, respectively) plus docetaxel versus ADT plus docetaxel alone.^23^, ^24^

Darolutamide is a structurally distinct and highly potent androgen receptor inhibitor with low blood–brain barrier penetration and limited potential for clinically relevant drug–drug interactions.^25^, ^26^, ^27^, ^28^, ^29^ In ARASENS, addition of darolutamide to ADT and docetaxel significantly reduced the risk of death by 32.5% versus placebo (hazard ratio [HR] 0.68; 95% confidence interval [CI] 0.57–0.80; *p* < 0.0001).^23^ The survival benefit was seen in patients with high‐volume disease and in those with high‐ or low‐risk disease, and the survival analysis was suggestive of benefit in the smaller subgroup with low‐volume disease.^30^ Darolutamide demonstrated a favorable safety profile, with low incidences of most treatment‐emergent adverse events (TEAEs) commonly associated with ARPI therapy and similar rates of discontinuations due to TEAEs versus placebo in clinical studies.^23^, ^31^, ^32^

Given the limited data on clinical outcomes in Asian patients with prostate cancer, including Japanese patients and especially those treated with docetaxel, it is important to understand whether the efficacy and safety in Japanese patients is likely to be the same or different from non‐Japanese patients. A phase 1 study of darolutamide in patients with metastatic CRPC showed no differences in safety or pharmacokinetics in Japanese versus non‐Japanese patients.^33^ In the phase 3 ARAMIS study in nonmetastatic CRPC, efficacy and safety benefits of darolutamide over placebo were consistent in Japanese patients versus the overall study population.^34^ Early treatment intensification with ADT plus an ARPI with docetaxel to delay progression and improve survival is becoming one of the new standards of care for patients with mHSPC. It is important to evaluate all available data to indicate whether this new treatment approach could be incorporated into clinical practice for Japanese patients. Therefore, we investigated efficacy and safety outcomes of treatment with darolutamide in combination with ADT and docetaxel in Japanese patients in ARASENS. A plain‐language summary of the findings is available in Supporting Document S1.

## 2 METHODS

ARASENS (ClinicalTrials.gov identifier NCT02799602) was a randomized, double‐blind, placebo‐controlled, phase 3 study conducted in 286 centers in 23 countries worldwide, including 44 centers in Japan. Full methods have been reported previously^23^ and are summarized briefly here.

Patients aged ≥18 years with mHSPC were eligible if they had Eastern Cooperative Oncology Group (ECOG) performance status 0 or 1 and were candidates for ADT and docetaxel. Patients were randomized 1:1 to oral darolutamide 600 mg twice daily or placebo, stratified by extent of disease (M1a, non‐regional lymph node metastases only; M1b, bone metastases ± lymph node metastases; or M1c, visceral metastases ± lymph node or bone metastases) and alkaline phosphatase (ALP) level (< or ≥ upper limit of normal [ULN]). All patients received standard of care with ADT and 6 cycles of docetaxel 75 mg/m^2^ every 3 weeks, with prednisone or equivalent at the investigator's discretion.

The primary endpoint was overall survival from randomization. Secondary endpoints were time to CRPC; time to pain progression; symptomatic skeletal event (SSE)‐free survival; time to first SSE; time to initiation of subsequent systemic antineoplastic therapy; time to worsening of disease‐related physical symptoms; time to initiation of opioid use for ≥7 or more consecutive days; and safety.

Efficacy was assessed in the full analysis set, which comprised all randomized patients available for analysis, assessed according to the treatment to which they were randomized. Secondary endpoints were tested with a hierarchical gatekeeping procedure, in the order described above, only if the primary endpoint and each preceding secondary endpoint in the hierarchy reached statistical significance. Differences between treatment groups were analyzed using a stratified log‐rank test, using randomization stratification factors. HRs (darolutamide vs. placebo) and 95% CIs were calculated using the Cox model stratified by randomization stratification factors. Two‐sided *p‐*values are reported for the overall population. Statistical analyses for the Japanese subgroup are exploratory. Safety assessments are reported descriptively for the safety analysis set, which comprised all patients who received at least one dose of darolutamide or placebo, assessed according to treatment received.

## 3 RESULTS

Between November 2016 and June 2018, 1306 patients were randomized globally, 1305 (darolutamide 651, placebo 654) of whom were available for analysis, including 148 patients in Japan (darolutamide 63, placebo 85). Baseline patient demographics and disease characteristics were similar in the two treatment groups in the Japanese subgroup (Table 1). These baseline characteristics were also generally comparable to those of the overall population, although Japanese patients had slightly older age, lower body mass index, and higher rates of de novo mHSPC, Gleason score ≥8 at diagnosis, and ECOG performance status 0.

**TABLE 1:** Patient demographics and baseline characteristics.

Characteristic	Japanese subgroup		Overall ARASENS population	
	Darolutamide + ADT + docetaxel *n* = 63	Placebo + ADT + docetaxel *n* = 85	Darolutamide + ADT + docetaxel *n* = 651	Placebo + ADT + docetaxel *n* = 654
Median age (range), years	73 (51–86)	70 (52–85)	67 (41–89)	67 (42–86)
Age group, *n* (%)				
<65 years	8 (12.7)	17 (20.0)	243 (37.3)	234 (35.8)
65–74 years	33 (52.4)	49 (57.6)	303 (46.5)	306 (46.8)
75–84 years	21 (33.3)	18 (21.2)	102 (15.7)	110 (16.8)
≥85 years	1 (1.6)	1 (1.2)	3 (0.5)	4 (0.6)
ECOG performance status, *n* (%)				
0	58 (92.1)	75 (88.2)	466 (71.6)	462 (70.6)
1	5 (7.9)	10 (11.8)	185 (28.4)	190 (29.1)
Body mass index, *n* (%)				
<20 kg/m^2^	14 (22.2)	12 (14.1)	45 (6.9)	34 (5.2)
20–<25 kg/m^2^	35 (55.6)	55 (64.7)	254 (39.0)	248 (37.9)
25–<30 kg/m^2^	12 (19.0)	15 (17.6)	240 (36.9)	254 (38.8)
≥30 kg/m^2^	2 (3.2)	3 (3.5)	108 (16.6)	116 (17.7)
Gleason score at initial diagnosis, *n* (%)				
<8	4 (6.3)	7 (8.2)	122 (18.7)	118 (18.0)
≥8	59 (93.7)	78 (91.8)	505 (77.6)	516 (78.9)
Metastatic stage at initial diagnosis, *n* (%)				
M1: distant metastasis	60 (95.2)	82 (96.5)	558 (85.7)	566 (86.5)
M0: no distant metastasis	3 (4.8)	3 (3.5)	86 (13.2)	82 (12.5)
Disease volume, *n* (%)^a^				
High	48 (76.2)	62 (72.9)	497 (76.3)	508 (77.7)
Low	15 (23.8)	23 (27.1)	154 (23.7)	146 (22.3)
Prognostic risk group, *n* (%)^b^				
High	50 (79.4)	67 (78.8)	452 (69.4)	460 (70.3)
Low	13 (20.6)	18 (21.2)	199 (30.6)	194 (29.7)
Metastatic stage at screening, *n* (%)				
M1a: non‐regional lymph node metastases only	2 (3.2)	1 (1.2)	23 (3.5)	16 (2.4)
M1b: bone metastases ± lymph node metastases	51 (81.0)	70 (82.4)	517 (79.4)	520 (79.5)
M1c: visceral metastases ± lymph node or bone metastases	10 (15.9)	14 (16.5)	111 (17.1)	118 (18.0)
Median serum prostate‐specific antigen (range), ng/mL^c^	24.7 (0.2–2648.8)	36.3 (0.1–2552.1)	30.3 (0.0–9219.0)	24.2 (0.0–11,947.0)
Median serum ALP (range), U/L^c^	137 (45–3986)	149 (57–4854)	148 (40–4885)	140 (36–7680)
ALP category, *n* (%)^c^				
<ULN	31 (49.2)	34 (40.0)	290 (44.5)	291 (44.5)
≥ULN	32 (50.8)	51 (60.0)	361 (55.5)	363 (55.5)

*Note*: Data for the overall population are from the *New England Journal of Medicine*, Smith MR, Hussain M, Saad F, et al., Darolutamide and Survival in Metastatic, Hormone‐Sensitive Prostate Cancer, Volume 386, Pages 1132–1142 Copyright © 2022 Massachusetts Medical Society. Reprinted with permission.

^a^ Disease volume classified according to CHAARTED: high volume = visceral metastases and/or ≥4 bone metastases with ≥1 beyond vertebral column and pelvis.^13^

^b^ Prognostic risk classified according to LATITUDE: high risk = two of Gleason score ≥8, ≥3 bone metastases, and presence of visceral metastases.^16^

^c^ Centrally assessed.

In the Japanese subgroup, the median treatment duration was longer for darolutamide versus placebo (43.3 vs. 15.4 months), consistent with the overall population (41.0 vs. 16.7 months). At the data cutoff date (October 25, 2021), the proportion of Japanese patients still receiving study treatment was higher for darolutamide (31 patients, 49.2%, ongoing) versus placebo (16 patients, 18.8%, ongoing), consistent with the overall population (darolutamide 299, 45.9%; placebo 125, 19.1%). Similar proportions of patients completed 6 cycles of docetaxel in the Japanese subgroup (darolutamide 52, 82.5%; placebo 70, 82.4%) and overall population (darolutamide 571, 87.6%; placebo 556, 85.5%). More patients required docetaxel dose reductions in the Japanese subgroup (darolutamide 33, 52.4%; placebo 39, 45.9%) versus the overall population (darolutamide 135, 20.7%; placebo 129, 19.8%), but the frequency was similar in the darolutamide and placebo groups in the Japanese subgroup. Use of granulocyte colony‐stimulating factor in patients treated with docetaxel was also similar in each treatment group in the overall population (darolutamide group 272/642 patients [42.4%] vs. placebo group 284/637 patients [44.6%]), occurring primarily after the first cycle of docetaxel (269/642 [41.9%] vs. 282/637 [44.3%]).

**FIGURE 1:**
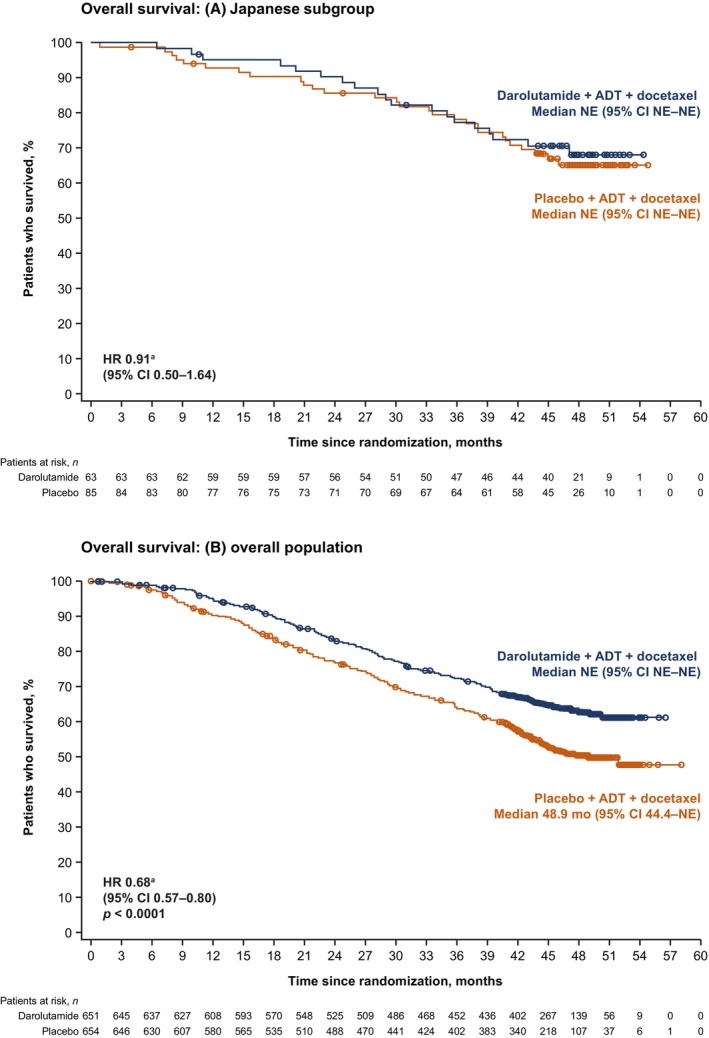
Overall survival in (A) the Japanese subgroup and (B) the overall population. ^a^HR stratified by extent of disease (M1a vs. M1b vs. M1c) and ALP level (< vs. ≥ ULN). Part (B) is from the *New England Journal of Medicine*, Smith MR, Hussain M, Saad F, et al., Darolutamide and Survival in Metastatic, Hormone‐Sensitive Prostate Cancer, Volume 386, Pages 1132–1142 Copyright © 2022 Massachusetts Medical Society. Reprinted with permission. NE, not estimable.

In the Japanese subgroup, the stratified HR for death was 0.91 (95% CI 0.50–1.64) for darolutamide in combination with ADT and docetaxel versus placebo plus ADT and docetaxel (Figure 1; Table 2). Similar to the overall population, most Japanese patients received subsequent life‐prolonging therapy, particularly ARPIs, with a higher proportion in the placebo versus darolutamide group (Table 3).

**TABLE 2:** Efficacy endpoints.

Endpoint	Japanese subgroup					Overall ARASENS population				
	Darolutamide + ADT + docetaxel *n* = 63		Placebo + ADT + docetaxel *n* = 85		HR (95% CI)^a^	Darolutamide + ADT + docetaxel *n* = 651		Placebo + ADT + docetaxel *n* = 654		HR (95% CI)^a^, ^b^
	Median time to event, months	Patients with event, *n* (%)	Median time to event, months	Patients with event, *n* (%)		Median time to event, months	Patients with event, *n* (%)	Median time to event, months	Patients with event, *n* (%)	
Overall survival	NE	19 (30)	NE	28 (33)	0.91 (0.50–1.64)	NE	229 (35)	48.9	304 (46)	0.68 (0.57–0.80) *p* < 0.0001
Time to mCRPC	NE	16 (25)	25.0	45 (53)	0.31 (0.17–0.55)	NE	225 (35)	19.1	391 (60)	0.36 (0.30–0.42) *p* < 0.001
Time to pain progression	46.1	23 (37)	NE	29 (34)	0.90 (0.51–1.58)	NE	222 (34)	27.5	248 (38)	0.79 (0.66–0.95) *p =* 0.01
SSE‐free survival	NE	22 (35)	45.0	34 (40)	0.72 (0.41–1.26)	51.2	257 (40)	39.7	329 (50)	0.61 (0.52–0.72) *p* < 0.001
Time to first SSE	NE	13 (21)	NE	13 (15)	1.15 (0.52–2.54)	NE	95 (15)	NE	108 (17)	0.71 (0.54–0.94) *p =* 0.02
Time to initiation of subsequent systemic antineoplastic therapy	NE	23 (37)	19.4	61 (72)	0.35 (0.21–0.57)	NE	219 (34)	25.3	395 (60)	0.39 (0.33–0.46) *p* < 0.001
Time to worsening of disease‐related physical symptoms	11.0	42 (67)	11.3	48 (56)	0.93 (0.60–1.43)	19.3	351 (54)	19.4	308 (47)	1.04 (0.89–1.22) NA

*Note*: Data for the overall population are from the *New England Journal of Medicine*, Smith MR, Hussain M, Saad F, et al., Darolutamide and Survival in Metastatic, Hormone‐Sensitive Prostate Cancer, Volume 386, Pages 1132–1142 (supplementary appendix) Copyright © 2022 Massachusetts Medical Society. Reprinted with permission.

Abbreviations: NA, not applicable (the preceding endpoint did not reach statistical significance; therefore this endpoint was not eligible for statistical analysis); NE, not estimable.

^a^ HR stratified by extent of disease (M1a vs. M1b vs. M1c) and alkaline phosphatase level (< vs. ≥ ULN).

^b^ Two‐sided *p‐*values.

**TABLE 3:** Subsequent life‐prolonging systemic antineoplastic therapy.

Patients with subsequent life‐prolonging systemic antineoplastic therapy, *n* (%)^a^	Japanese subgroup		Overall ARASENS population	
	Darolutamide + ADT + docetaxel *n* = 27^a^	Placebo + ADT + docetaxel *n* = 66^a^	Darolutamide + ADT + docetaxel *n* = 315^a^	Placebo + ADT + docetaxel *n* = 495^a^
Any	19 (70.4)	56 (84.8)	179 (56.8)	374 (75.6)
Abiraterone acetate	11 (40.7)	26 (39.4)	112 (35.6)	232 (46.9)
Enzalutamide	9 (33.3)	38 (57.6)	48 (15.2)	136 (27.5)
Cabazitaxel	10 (37.0)	16 (24.2)	57 (18.1)	89 (18.0)
Docetaxel	5 (18.5)	8 (12.1)	46 (14.6)	89 (18.0)
Radium‐223	8 (29.6)	10 (15.2)	19 (6.0)	34 (6.9)
Sipuleucel‐T	0	0	4 (1.3)	10 (2.0)
Lutetium‐177 PSMA	0	0	2 (0.6)	7 (1.4)
Apalutamide	0	0	2 (0.6)	2 (0.4)

*Note*: Data for the overall population are from the *New England Journal of Medicine*, Smith MR, Hussain M, Saad F, et al., Darolutamide and Survival in Metastatic, Hormone‐Sensitive Prostate Cancer, Volume 386, Pages 1132–1142 (supplementary appendix) Copyright © 2022 Massachusetts Medical Society. Reprinted with permission.

Abbreviation: PSMA, prostate‐specific membrane antigen.

^a^ The denominators are the number of patients who entered active or long‐term (survival) follow‐up, plus one patient who did not enter follow‐up but received subsequent therapy. Patients could receive more than one subsequent therapy.

**FIGURE 2:**
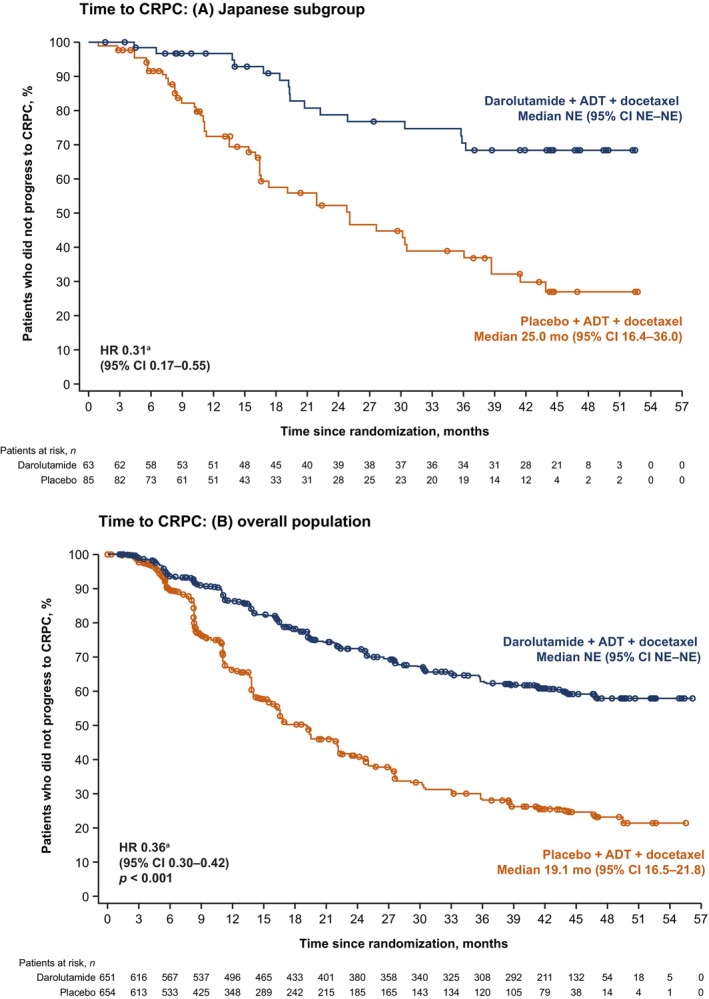
Time to CRPC in (A) the Japanese subgroup and (B) the overall population. ^a^HR stratified by extent of disease (M1a vs. M1b vs. M1c) and ALP level (< vs. ≥ ULN). Part (B) is from the *New England Journal of Medicine*, Smith MR, Hussain M, Saad F, et al., Darolutamide and Survival in Metastatic, Hormone‐Sensitive Prostate Cancer, Volume 386, Pages 1132–1142 Copyright © 2022 Massachusetts Medical Society. Reprinted with permission.

Consistent with the overall population, a strong treatment benefit of delaying time to CRPC versus placebo was observed in the Japanese subgroup (HR 0.31; 95% CI 0.17–0.55; Table 2, Figure 2). Darolutamide also showed trends versus placebo for delaying time to pain progression (HR 0.90; 95% CI 0.51–1.58) and time to initiation of subsequent systemic antineoplastic therapy (HR 0.35; 95% CI 0.21–0.57) in the Japanese subgroup, similar to the overall population (Table 2).

Despite longer treatment with darolutamide versus placebo (43.3 vs. 15.4 months), the incidences of TEAEs of any grade and severe TEAEs (grade ≥3) were generally similar between treatment groups in the Japanese subgroup (Table 4). The incidence of TEAEs leading to treatment discontinuation was low in both groups. The safety profile in the Japanese subgroup was generally similar to that of the overall ARASENS population, with a few exceptions. In the Japanese subgroup, both treatment groups had a higher incidence of grade 4 TEAEs and the darolutamide group had a higher incidence of serious TEAEs compared with the overall ARASENS population. Febrile neutropenia was the only individual serious TEAE that occurred in >5% of patients and was reported in more placebo recipients than darolutamide recipients (14% vs. 8%).

**TABLE 4:** Treatment‐emergent adverse event (TEAE) overview.

Patients, *n* (%)	Japanese subgroup		Overall ARASENS population	
	Darolutamide + ADT + docetaxel *n* = 63	Placebo + ADT + docetaxel *n* = 85	Darolutamide + ADT + docetaxel *n* = 652	Placebo + ADT + docetaxel *n* = 650
Any TEAE	63 (100.0)	85 (100.0)	649 (99.5)	643 (98.9)
Worst severity				
Grade 1	0	2 (2.4)	28 (4.3)	35 (5.4)
Grade 2	5 (7.9)	8 (9.4)	162 (24.8)	169 (26.0)
Grade 3	15 (23.8)	19 (22.4)	248 (38.0)	232 (35.7)
Grade 4	40 (63.5)	55 (64.7)	183 (28.1)	181 (27.8)
Grade 5	3 (4.8)	1 (1.2)	27 (4.1)	26 (4.0)
Serious TEAE	37 (58.7)	39 (45.9)	292 (44.8)	275 (42.3)
TEAE leading to permanent treatment discontinuation				
Darolutamide/placebo	11 (17.5)	9 (10.6)	88 (13.5)	69 (10.6)
Docetaxel	9 (14.3)	12 (14.1)	52 (8.0)	67 (10.3)

*Note*: Data for the overall population are from the *New England Journal of Medicine*, Smith MR, Hussain M, Saad F, et al., Darolutamide and Survival in Metastatic, Hormone‐Sensitive Prostate Cancer, Volume 386, Pages 1132–1142 Copyright © 2022 Massachusetts Medical Society. Reprinted with permission.

Incidences of the most frequently reported TEAEs were generally similar between treatment groups in the Japanese subgroup (Table 5). Certain TEAEs known to be associated with docetaxel therapy, including decreased neutrophil counts and peripheral edema, were higher in Japanese patients than in the overall population. The incidence of the most frequently reported TEAEs was highest during the time overlapping with docetaxel treatment. The incidence of TEAEs commonly associated with ARPI therapy was generally low in both treatment groups in the Japanese subgroup and aligned with the overall population (Table 6). In the Japanese subgroup, rash, vasodilation and flushing, cardiac disorders, falls, and bone fractures were the most frequently reported AEs of interest. Fatigue, vasodilatation and flushing, rash, and diabetes mellitus were the most frequently reported AEs of interest in the overall population.

**TABLE 5:** Most frequent treatment‐emergent adverse events (TEAEs).^a^

TEAEs	Japanese subgroup				Overall ARASENS population			
	Darolutamide + ADT + docetaxel *n* = 63		Placebo + ADT + docetaxel *n* = 85		Darolutamide + ADT + docetaxel *n* = 652		Placebo + ADT + docetaxel *n* = 650	
	*n* (%)	EAIR^b^	*n* (%)	EAIR^b^	*n* (%)	EAIR^b^	*n* (%)	EAIR^b^
Any grade								
Neutrophil count decreased	40 (63.5)	22.3	54 (63.5)	33.1	170 (26.1)	9.8	155 (23.8)	12.9
White blood cell count decreased	39 (61.9)	21.8	43 (50.6)	26.4	155 (23.8)	9.0	143 (22.0)	11.9
Alopecia	37 (58.7)	20.7	54 (63.5)	33.1	264 (40.5)	15.3	264 (40.6)	22.0
Edema peripheral	26 (41.3)	14.5	35 (41.2)	21.5	173 (26.5)	10.0	169 (26.0)	14.1
Malaise	23 (36.5)	12.8	38 (44.7)	23.3	57 (8.7)	3.3	66 (10.2)	5.5
Anemia	23 (36.5)	12.8	20 (23.5)	12.3	181 (27.8)	10.5	163 (25.1)	13.6
Dysgeusia	23 (36.5)	12.8	18 (21.2)	11.0	69 (10.6)	4.0	80 (12.3)	6.7
Constipation	22 (34.9)	12.3	27 (31.8)	16.6	147 (22.5)	8.5	130 (20.0)	10.8
Diarrhea	22 (34.9)	12.3	23 (27.1)	14.1	167 (25.6)	9.6	156 (24.0)	13.0
Decreased appetite	21 (33.3)	11.7	17 (20.0)	10.4	121 (18.6)	7.0	85 (13.1)	7.1
Peripheral sensory neuropathy	19 (30.2)	10.6	30 (35.3)	18.4	65 (10.0)	3.8	67 (10.3)	5.6
Dry skin	18 (28.6)	10.1	11 (12.9)	6.7	47 (7.2)	2.7	35 (5.4)	2.9
Pyrexia	15 (23.8)	8.4	18 (21.2)	11.0	86 (13.2)	5.0	90 (13.8)	7.5
Nasopharyngitis	14 (22.2)	7.8	9 (10.6)	5.5	45 (6.9)	2.6	45 (6.9)	3.7
Arthralgia	13 (20.6)	7.3	19 (22.4)	11.6	178 (27.3)	10.3	174 (26.8)	14.5
Nausea	13 (20.6)	7.3	18 (21.2)	11.0	115 (17.6)	6.6	133 (20.5)	11.1
Pruritus	13 (20.6)	7.3	12 (14.1)	7.4	45 (6.9)	2.6	52 (8.0)	4.3
Upper respiratory tract infection	12 (19.0)	6.7	12 (14.1)	7.4	54 (8.3)	3.1	47 (7.2)	3.9
Aspartate aminotransferase increased	12 (19.0)	6.7	11 (12.9)	6.7	91 (14.0)	5.3	68 (10.5)	5.7
Back pain	10 (15.9)	5.6	15 (17.6)	9.2	123 (18.9)	7.1	123 (18.9)	10.2
Alanine aminotransferase increased	10 (15.9)	5.6	11 (12.9)	6.7	102 (15.6)	5.9	84 (12.9)	7.0
Febrile neutropenia	9 (14.3)	5.0	17 (20.0)	10.4	51 (7.8)	2.9	48 (7.4)	4.0
Nail discoloration	7 (11.1)	3.9	13 (15.3)	8.0	48 (7.4)	2.8	51 (7.8)	4.2
Insomnia	6 (9.5)	3.4	16 (18.8)	9.8	74 (11.3)	4.3	81 (12.5)	6.7
Grade 3 or 4								
Neutrophil count decreased	39 (61.9)		51 (60.0)		151 (23.2)		140 (21.5)	
White blood cell count decreased	35 (55.6)		36 (42.4)		110 (16.9)		97 (14.9)	
Febrile neutropenia	9 (14.3)		17 (20.0)		51 (7.8)		48 (7.4)	
Anemia	5 (7.9)		7 (8.2)		31 (4.8)		33 (5.1)	
Neutropenia	5 (7.9)		5 (5.9)		56 (8.6)		68 (10.5)	
Alanine aminotransferase increased	4 (6.3)		2 (2.4)		18 (2.8)		11 (1.7)	

*Note*: Data for the overall population are from the *New England Journal of Medicine*, Smith MR, Hussain M, Saad F, et al., Darolutamide and Survival in Metastatic, Hormone‐Sensitive Prostate Cancer, Volume 386, Pages 1132–1142 and supplementary appendix Copyright © 2022 Massachusetts Medical Society. Reprinted with permission.

^a^ The table includes all TEAEs occurring in ≥15% and grade 3 or 4 TEAEs in ≥5% of Japanese patients in either treatment group.

^b^ Exposure‐adjusted incidence rate (EAIR) is the number of patients with a TEAE divided by the darolutamide/placebo treatment duration of all patients, expressed in 100 patient‐years.

**TABLE 6:** Treatment‐emergent adverse events (TEAEs) of special interest associated with ARPI therapy.

TEAEs of special interest	Japanese subgroup				Overall ARASENS population			
	Darolutamide + ADT + docetaxel *n* = 63		Placebo + ADT + docetaxel *n* = 85		Darolutamide + ADT + docetaxel *n* = 652		Placebo + ADT + docetaxel *n* = 650	
	*n* (%)	EAIR^a^	*n* (%)	EAIR^a^	*n* (%)	EAIR^a^	*n* (%)	EAIR^a^
Fatigue	3 (4.8)	1.7	2 (2.4)	1.2	216 (33.1)	12.5	214 (32.9)	17.8
Vasodilatation and flushing	8 (12.7)	4.5	12 (14.1)	7.4	133 (20.4)	7.7	141 (21.7)	11.7
Rash^b^	13 (20.6)	7.3	14 (16.5)	8.6	108 (16.6)	6.2	88 (13.5)	7.3
Diabetes mellitus and hyperglycemia	2 (3.2)	1.1	7 (8.2)	4.3	99 (15.2)	5.7	93 (14.3)	7.7
Hypertension^c^	4 (6.3)	2.2	4 (4.7)	2.5	89 (13.7)	5.1	60 (9.2)	5.0
Cardiac disorder	7 (11.1)	3.9	2 (2.4)	1.2	71 (10.9)	4.1	76 (11.7)	6.3
Cardiac arrhythmia^c^	3 (4.8)	1.7	2 (2.4)	1.2	52 (8.0)	3.0	55 (8.5)	4.6
Coronary artery disorder^c^	3 (4.8)	1.7	0	0	19 (2.9)	1.1	13 (2.0)	1.1
Heart failure^c^	1 (1.6)	0.6	0	0	4 (0.6)	0.2	13 (2.0)	1.1
Bone fracture^d^	6 (9.5)	3.4	4 (4.7)	2.5	49 (7.5)	2.8	33 (5.1)	2.7
Falls, including accident	7 (11.1)	3.9	2 (2.4)	1.2	43 (6.6)	2.5	30 (4.6)	2.5
Mental‐impairment disorder^c^	3 (4.8)	1.7	1 (1.2)	0.6	23 (3.5)	1.3	15 (2.3)	1.2
Weight decreased	2 (3.2)	1.1	4 (4.7)	2.5	22 (3.4)	1.3	35 (5.4)	2.9
Depressed‐mood disorder^c^	0	0	1 (1.2)	0.6	21 (3.2)	1.2	24 (3.7)	2.0
Breast disorders/gynecomastia^c^	1 (1.6)	0.6	0	0	21 (3.2)	1.2	10 (1.5)	0.8
Cerebral ischemia	1 (1.6)	0.6	1 (1.2)	0.6	8 (1.2)	0.5	8 (1.2)	0.7

*Note*: Data for the overall population are from the *New England Journal of Medicine*, Smith MR, Hussain M, Saad F, et al., Darolutamide and Survival in Metastatic, Hormone‐Sensitive Prostate Cancer, Volume 386, Pages 1132–1142 supplementary appendix Copyright © 2022 Massachusetts Medical Society. Reprinted with permission.

^a^ Exposure‐adjusted incidence rate (EAIR) is the number of patients with a TEAE divided by the darolutamide/placebo treatment duration of all patients, expressed in 100 patient‐years.

^b^ This category combines the following Medical Dictionary for Regulatory Activities (MedDRA) terms: rash, maculopapular rash, drug eruption, pruritic rash, erythematous rash, macular rash, papular rash, follicular rash, pustular rash, and vesicular rash.

^c^ This category is a MedDRA High‐Level Group Term.

^d^ Excluding pathologic fractures.

## 4 DISCUSSION

In patients with mHSPC, there remains a high unmet need for effective treatment to increase survival and delay disease progression to metastatic CRPC. With ADT alone, survival is only 3–6 years, and castration resistance develops within 1–3 years.^35^, ^36^, ^37^, ^38^, ^39^, ^40^, ^41^ The combination of ADT with docetaxel or an ARPI can improve outcomes.^38^, ^39^, ^40^, ^41^ The ARASENS study demonstrated the OS benefit of combining darolutamide with ADT and docetaxel versus placebo plus ADT and docetaxel. As is common for phase 3 global studies, ARASENS was not powered to allow statistical analysis of subgroups, such as the Japanese subpopulation, and the findings in this subgroup analysis are descriptive only; nevertheless, we believe that, with the number of Japanese participants in ARASENS (*n* = 148, 11.3% of the total ARASENS population), the findings in this subpopulation provide an indication of treatment outcomes that might be expected in clinical practice to help guide treatment decisions in Japanese patients. In addition, because there are few prospective data on treatment including docetaxel in Japanese patients with mHSPC, our analyses should be meaningful for medical doctors and other healthcare professionals. In the ARASENS Japanese subpopulation, despite being more likely to have features of high‐risk disease (>90% of patients had de novo mHSPC and Gleason score ≥8 at diagnosis) compared with the overall population, the data were suggestive of benefits with darolutamide over placebo for many of the clinically relevant efficacy endpoints, consistent with those observed in the overall population. Given that development of castration resistance is associated with reduced survival,^1^, ^35^ prolonging the time to CRPC is an important and clinically relevant endpoint. Among darolutamide‐treated patients with mHSPC, the time to CRPC was prolonged versus placebo in both the Japanese subgroup and the overall ARASENS population.

The combination of darolutamide with ADT and docetaxel was generally well tolerated in Japanese patients. Despite being on average older with lower body mass index than the overall population, factors that might be expected to affect drug absorption and tolerability,^42^, ^43^ most Japanese patients (>82%) were able to receive the full 6 cycles of docetaxel and maintain long‐term darolutamide treatment with low discontinuation rates, consistent with the overall population. The incidences of most TEAEs were generally similar between treatment groups in Japanese patients, although the frequency of serious TEAEs was proportionally higher with darolutamide versus placebo (37 patients, 58.7%, vs. 39 patients, 45.9%). However, the only individual serious TEAE that occurred in >5% of patients was febrile neutropenia, which was reported in more placebo recipients (14%) than darolutamide recipients (8%). The longer median treatment duration with darolutamide (43.3 months) versus placebo (15.4 months), might have provided more time for patients on darolutamide to experience TEAEs, including serious adverse events. However, numbers are small, making it inappropriate to draw firm conclusions from these findings.

As in the overall population, many of the most common TEAEs were those known to be associated with docetaxel treatment, such as neutropenia. The incidences of these events tended to be higher in Japanese patients, which might be related to ethnicity; high frequencies of such events have been seen in previous studies of docetaxel in Japanese patients.^44^, ^45^, ^46^, ^47^ The proportions of patients with falls and cardiac disorders were numerically higher in the darolutamide group (seven patients [11.1%] each) versus the placebo group (two patients [2.4%] each) in the Japanese subgroup; this difference may be an artifact of the small subgroup size, but might also be related to age, as the proportion of patients aged 75–84 years was higher in the darolutamide versus placebo group (33.3% vs. 21.2%). As recommended by the International Society of Geriatric Oncology, elderly patients should have a comprehensive geriatric assessment, and patients who are deemed to be fit should be treated in the same way as younger patients, whereas frail patients may need an adapted approach.^48^ Additionally, the higher incidence of falls and cardiac disorders might be related to the longer treatment period of darolutamide versus placebo, because the differences in exposure‐adjusted incidence rates are less prominent. Patients should be proactively advised about TEAEs when using this combination, particularly TEAEs commonly associated with docetaxel, which typically occur in the first 6 months (during the period of docetaxel therapy) and reduce in frequency thereafter.

Interpretation of the findings in Japanese patients must take into account that the study was not powered for this subgroup analysis, and results are descriptive only. Nevertheless, darolutamide showed trends of improved efficacy over placebo, with similar tolerability and no new safety concerns, which support the use of darolutamide in combination with ADT and docetaxel in Japanese patients with mHSPC. These results are consistent with the overall ARASENS population, suggesting that darolutamide in combination with ADT and docetaxel should be considered as one of the new standards of care for the treatment of patients with mHSPC, including Japanese patients.

## AUTHOR CONTRIBUTIONS

**Motohide Uemura:** Conceptualization (equal); data curation (equal); formal analysis (equal); investigation (equal); writing – review and editing (equal). **Hiroaki Kikukawa:** Data curation (supporting); formal analysis (supporting); investigation (supporting); writing – review and editing (supporting). **Yasuhiro Hashimoto:** Data curation (supporting); formal analysis (supporting); investigation (supporting); writing – review and editing (supporting). **Hiroji Uemura:** Data curation (supporting); formal analysis (supporting); investigation (supporting); writing – review and editing (supporting). **Atsushi Mizokami:** Data curation (supporting); formal analysis (supporting); investigation (supporting); writing – review and editing (supporting). **Masashi Kato:** Data curation (supporting); formal analysis (supporting); investigation (supporting); writing – review and editing (supporting). **Hisashi Matsushima:** Data curation (supporting); formal analysis (supporting); investigation (supporting); writing – review and editing (supporting). **Takeo Kosaka:** Data curation (supporting); formal analysis (supporting); investigation (supporting); writing – review and editing (supporting). **Motonobu Nakamura:** Data curation (supporting); formal analysis (supporting); investigation (supporting); writing – review and editing (supporting). **Satoshi Fukasawa:** Data curation (supporting); formal analysis (supporting); investigation (supporting); writing – review and editing (supporting). **Matthew R. Smith:** Conceptualization (lead); data curation (supporting); formal analysis (supporting); investigation (supporting); methodology (supporting); writing – review and editing (supporting). **Bertrand Tombal:** Conceptualization (equal); data curation (supporting); formal analysis (supporting); investigation (supporting); methodology (supporting); writing – review and editing (supporting). **Maha Hussain:** Conceptualization (equal); data curation (supporting); formal analysis (supporting); investigation (supporting); methodology (supporting); writing – review and editing (supporting). **Fred Saad:** Conceptualization (equal); data curation (supporting); formal analysis (supporting); investigation (supporting); methodology (supporting); writing – review and editing (supporting). **Karim Fizazi:** Conceptualization (equal); data curation (supporting); formal analysis (supporting); investigation (supporting); methodology (supporting); writing – review and editing (supporting). **Cora N. Sternberg:** Conceptualization (equal); data curation (supporting); formal analysis (supporting); investigation (supporting); methodology (supporting); writing – review and editing (supporting). **E. David Crawford:** Conceptualization (equal); data curation (supporting); formal analysis (supporting); investigation (supporting); methodology (supporting); writing – review and editing (supporting). **Haruka Kakiuchi:** Formal analysis (supporting); project administration (supporting); supervision (supporting); writing – review and editing (supporting). **Masanao Akiyama:** Formal analysis (supporting); project administration (supporting); supervision (supporting); writing – review and editing (supporting). **Rui Li:** Data curation (supporting); formal analysis (supporting); methodology (supporting); validation (supporting); writing – review and editing (supporting). **Iris Kuss:** Conceptualization (supporting); data curation (supporting); formal analysis (equal); methodology (supporting); validation (supporting); writing – review and editing (supporting). **Heikki Joensuu:** Data curation (supporting); formal analysis (supporting); writing – review and editing (supporting). **Hiroyoshi Suzuki:** Conceptualization (supporting); data curation (equal); formal analysis (equal); investigation (equal); writing – review and editing (equal).

## FUNDING INFORMATION

The ARASENS study was supported by Bayer and Orion Pharma.

## CONFLICT OF INTEREST STATEMENT

Motohide Uemura, Hiroaki Kikukawa, Yasuhiro Hashimoto, and Hiroji Uemura have no conflict of interest; Atsushi Mizokami received fees, honoraria, and remuneration from Bayer; Masashi Kato has no conflict of interest; Hisashi Matsushima received fees and honoraria from Janssen; Takeo Kosaka, Motonobu Nakamura, and Satoshi Fukasawa have no conflict of interest; Matthew R. Smith received fees, honoraria, and remuneration from Bayer; Bertrand Tombal received fees, honoraria, and remuneration from Astellas, Bayer, Janssen, and Sanofi; Maha Hussain received fees and honoraria from Astellas and Bayer, and research funds paid to her institution from Bayer; Fred Saad received fees and honoraria from Astellas, Bayer, Janssen, and Sanofi, and research funding paid to his institution from Astellas, Bayer, Janssen, and Sanofi; Karim Fizazi received fees and honoraria from Astellas, Bayer, Janssen, and Sanofi; Cora N. Sternberg received fees and honoraria from Astellas and Bayer; E. David Crawford received fees, honoraria, and remuneration from Bayer; Haruka Kakiuchi, Masanao Akiyama, Rui Li, and Iris Kuss received remuneration from Bayer (employees); Heikki Joensuu received remuneration from Orion Pharma, is Chair of the Scientific Advisory Board of Neutron Therapeutics, and owns stock in Orion Pharma and Sartar Therapeutics; Hiroyoshi Suzuki received fees, honoraria, and remuneration from Bayer and Sanofi. The study was designed under the responsibility of Bayer and Orion Pharma, in conjunction with the protocol steering committee; the study was funded by Bayer and Orion Pharma; darolutamide was provided by Bayer; Bayer collected and analyzed the data and contributed to the interpretation of the study. All authors had full access to all of the data in the study and had final responsibility for the decision to submit for publication.

## ETHICS STATEMENT

Approval of the research protocol by an institutional review board (IRB): The independent ethics committee or IRB for each participating institution approved the ARASENS study protocol. In Japan, the relevant IRBs were Jikei University Hospital Group IRB, IRB of Juntendo University Hospital, JCHO Tokyo Shinjuku Medical Center IRB, IRB of Yokohama City University Medical Center, IRB of Chiba University Hospital, National Cancer Center Hospital East IRB, IRB of Osaka International Cancer Institute, Kindai University Hospital IRB, IRB of Kagawa University Hospital, Tohoku University Hospital IRB, IRB of NHO Shikoku Cancer Center, IRB of NHO Hokkaido Cancer Center, Kobe University Hospital IRB, Osaka City University Hospital IRB, Toho University Sakura Medical Center IRB, IRB of Hamamatsu University Hospital, The University of Tokyo Hospital IRB, Tottori University Hospital IRB, Saiseikai Imperial Gift Foundation Inc Central IRB, IRB of Kanazawa University Hospital, Chiba Cancer Center IRB, IRB of Nagoya University Hospital, Osaka University Hospital IRB, Kyorin University Hospital IRB, IRB of the Cancer Institute Hospital of JFCR, IRB of Nippon Medical School Hospital, IRB of Mie University Hospital, NHO Kyushu Cancer Center IRB, IRB of Yamaguchi University Hospital, Nara Medical University Hospital IRB, IRB of University of Miyazaki Hospital, Jichi Medical University Hospital IRB, NHO Kumamoto Medical Center IRB, Ishikawa Prefectural Central Hospital IRB, IRB of Okayama University Hospital, IRB of TMPH, IRB of Keio University Hospital, Kanagawa Cancer Center IRB, IRB of Hirosaki University School of Medicine & Hospital, IRB of Wakayama Medical University Hospital, Nagasaki University Hospital IRB, Tokushima University Hospital IRB, Asahi General Hospital IRB, NHO Tokyo Medical Center IRB, and Gifu Prefectural General Medical Center IRB. Informed consent: all patients provided written informed consent. Registry and registration number of the trial: ClinicalTrials.gov identifier NCT02799602. Permission to reproduce material from other sources: Data on the overall ARASENS population in Figures 1 and 2 and Tables 1, 2, 3, 4, 5, 6 are from Smith MR, Hussain M, Saad F, et al. Darolutamide and survival in metastatic, hormone‐sensitive prostate cancer. *N Engl J Med*. 386:1132–1142 Copyright © 2022 Massachusetts Medical Society. Reprinted with permission.

## CLINICAL TRIAL REGISTRATION

ClinicalTrials.gov identifier NCT02799602.

## Supporting information


**Data S1:** Supporting information.

## Data Availability

Availability of the data underlying this publication will be determined according to Bayer's commitment to the EFPIA/PhRMA “Principles for responsible clinical trial data sharing.” This pertains to scope, timepoint and process of data access. As such, Bayer commits to sharing upon request from qualified scientific and medical researchers patient‐level clinical trial data, study‐level clinical trial data, and protocols from clinical trials in patients for medicines and indications approved in the United States (US) and European Union (EU) as necessary for conducting legitimate research. This applies to data on new medicines and indications that have been approved by the EU and US regulatory agencies on or after January 01, 2014. Interested researchers can use www.vivli.org to request access to anonymized patient‐level data and supporting documents from clinical studies to conduct further research that can help advance medical science or improve patient care. Information on the Bayer criteria for listing studies and other relevant information is provided in the member section of the portal. Data access will be granted to anonymized patient‐level data, protocols and clinical study reports after approval by an independent scientific review panel. Bayer is not involved in the decisions made by the independent review panel. Bayer will take all necessary measures to ensure that patient privacy is safeguarded.
