# Supplementary material for: Darolutamide in Japanese patients with metastatic hormone‐sensitive prostate cancer: Phase 3 ARASENS subgroup analysis
Source: Cancer Med. 2024 Nov 11;13(21):e70029. doi: 10.1002/cam4.70029 (PMC11552649; doi:10.1002/cam4.70029)
Supplement: Supplementary file 1 — Data S1: Supporting information. [file CAM4-13-e70029-s001.docx]

**Supporting Doc S1:**

**Plain language summary**

Prostate cancer usually depends on the hormone testosterone in order to grow. Androgen-deprivation therapy (or ADT) is a common treatment to reduce testosterone levels, which in turn slows the growth of the cancer. Metastatic hormone-sensitive prostate cancer (or mHSPC) is a type of prostate cancer that has spread to other parts of the body (“metastatic”) but still responds to ADT (“hormone sensitive”). However, the cancer will eventually start to grow again despite treatment with ADT – this stage of the cancer is referred to as metastatic castration-resistant prostate cancer (or mCRPC) and the outlook is poor for patients. To help patients live as long as possible and delay development of mCRPC, ADT is often combined with other kinds of treatment, such as docetaxel, which is a type of chemotherapy.

Darolutamide is an oral treatment that helps prevent testosterone from making prostate cancer grow. The ARASENS trial was designed to study whether patients with mHSPC lived longer if they were treated with darolutamide in combination with ADT and docetaxel, compared with patients who received ADT and docetaxel with placebo (sugar pill). ARASENS was a large, international study involving 1306 randomized patients, with data available for analysis from 1305 of these patients. The results showed that treatment with darolutamide in combination with ADT and docetaxel lowered the risk of death by 32.5% compared with placebo in combination with ADT and docetaxel. Because some treatments may affect Japanese and non-Japanese patients differently, we studied the 148 Japanese patients who took part in ARASENS; 63 of these patients received darolutamide, and 85 received placebo. The Japanese patients in ARASENS were slightly older, had a lower body mass index, and were more likely to have disease features indicating poor outlook compared with the overall trial participants. Japanese patients treated with darolutamide in combination with ADT and docetaxel tended to live longer than those treated with placebo in combination with ADT and docetaxel. The rates of medical problems (or “adverse events”) after initiation of treatment were similar regardless of whether patients were receiving darolutamide or placebo, and few patients had to stop study treatment because of these adverse events.

In conclusion, consistent with the findings in all ARASENS trial participants, Japanese patients treated with darolutamide tended to live longer than those treated with placebo, in combination with ADT and docetaxel. Furthermore, there were no new safety concerns with this combination in Japanese patients.
